# Bibliography

**Published:** 1910-01-15

**Authors:** 


### BIBLIOGRAPHY.

HISTORY OF DENTAL SURGERY. Edited by Charles R. E. Koch, D.D.S., and published by the National Art Publishing Co. of Chicago in two large volumes, has just come from the printers. The first volume of nearly 1200 pages contains a concise history of the development of dentistry from Egyptian and Grecian antiquity down to the present day, written by the Editor Dr. Koch. Then other contributors trace the development of the various subjects of professional interest; Edmund Noyes, that of Operative Dentistry; H. L. Ambler, Prosthodontia from early Chinese to modern crown and bridge work; S. H. Guilford, Orthodontia; W. H. Trueman, Dental Journals; E. C. Mills, Dental Literature; C. R. E. Koch and T. W. Brophy, Oral Surgery. The rest of this volume is given up to statistical statements as to the work of the Dental Colleges, number of graduates and where the various schools are located; the various educational organizations and their influences, etc. Then the subject of laws and legislations in various States is very comprehensively treated, statistically and historically. Dental Associations, Fraternal organizations and all International movements for professional advancement are given very careful consideration. The volume is beautifully printed and illustrated with many most valuable pictures of men, institutions, book plates, instruments and other matters of historic interest and great value. No such work has heretofore been attempted and the editors and publishers deserve the greatest commendations for gathering together in so compact and yet comprehensive a form the material which in the future will be of great value as a matter of reference and which even now is of great interest.

The second volume is edited almost entirely by Dr. Burton Lee Thorpe and is given up to biographic and autobiographic sketches of eminent men who have had much to do with the making of the profession in America. These sketches have been for several years appearing in the Dental Review first and more recently in the Dental Brief. As our readers are more or less familiar with them they need no comment here; but we are very glad to have them put into book form for more convenient reference. The history of our profession would be of little value without a history of the men most active in its making and Dr. Thorpe has done his work so well that the profession owe him a debt of gratitude.

We of course have not had the time to carefully review this entire work, but we have been delightfully surprised to find so much of interest in those subjects we have selected for examination. It is a work every dentist, who loves his profession, and who feels any obligation to the men who made it, should possess. The entire work shows evidence of much thought and work, some of which might be criticised and we sincerely trust the profession may extend its editors and publishers adequate appreciation.

